# Factors Associated with Cytomegalovirus Reactivation Following Allogeneic Hematopoietic Stem Cell Transplantation: Human Leukocyte Antigens Might Be Among the Risk Factors

**DOI:** 10.4274/Tjh.2013.0244

**Published:** 2014-09-05

**Authors:** Kadir Acar1, Şahika Zeynep Akı, Zübeyde Nur Özkurt, Gülendam Bozdayı, Seyyal Rota, Gülsan Türköz Sucak

**Affiliations:** 1 Gazi University Faculty of Medicine, Department of Hematology, Ankara, Turkey; 2 Gazi University Faculty of Medicine, Department of Microbiology, Ankara, Turkey

**Keywords:** Cytomegalovirus reactivation, Human leukocyte antigens, allogeneic stem cell transplantation, graft-versus-host disease, prognosis, CMV scoring index

## Abstract

**Objective:** Cytomegalovirus (CMV) is a significant cause of morbidity and mortality in allogeneic hematopoietic stem cell transplantation (AHSCT) recipients. Current practice includes prophylactic and preemptive treatment modalities, which have risks, side effects, and costs of their own. There is no established risk scoring system that applies to all patients. We aimed to investigate the risk factors for CMV reactivation in AHSCT recipients.

**Materials and Methods:** We retrospectively analyzed the risk factors for CMV reactivation in 185 consequent AHSCT recipients transplanted between September 2003 and December 2009 at the Stem Cell Transplantation Unit of Gazi University. Besides the standard transplant-related parameters, HLA antigens were also included among the variables analyzed.

**Results:** Despite the very high rate of donor (94.6%) and recipient (100%) seropositivity, which are the so-called major risk factors in previous reports, our reactivation rate was much lower, with a frequency of 24.9%. The underlying disease, sex, conditioning regimen, and presence of antithymocyte globulin or fludarabine in the conditioning regimen had no impact on reactivation rate. CMV reactivation was significantly more frequent in recipients with graft-versus-host disease (GVHD) compared to those without GVHD (p<0.0001). CMV reactivation was significantly more frequent (p<0.05) in patients with HLA-B14, HLA-DRB1*01, and HLA-DRB1*13 antigens and less frequent in recipients with HLA-A11 and HLA-DRB1*04 antigens (p<0.05).

**Conclusion:** Universal risk factors/scores that apply to all transplant recipients are required for tailored prophylaxis and/or treatment strategies for CMV reactivation. Uncovering the role of genetic factors, including HLA antigens, as possible risk factors might lead the way to risk-adaptive strategies for adoptive cellular therapy and/or vaccination.

## OZET

**Amaç:** Allojeneik kök hücre nakli (AKHN) alıcılarında sitomegalovirus (CMV) önemli mortalite ve morbidite nedenidir. Mevcut uygulama profilaksi ve önleyici tedavi yöntemleridir ki, bunun da riskleri, yan etkileri ve maliyeti vardır. Öte yandan tüm hastalar için geçerli bir yerleşik risk puanlama sistemi yoktur. Bu çalışmada, AKHN yapılan hastalarda CMV reaktivasyonu için risk faktörlerini araştırmayı amaçladık.

**Gereç ve Yöntemler:** Gazi Üniversitesi Kök Hücre Nakil Ünitesi’nde Eylül 2003 ve Aralık 2009 tarihleri ararsında AKHN yapılan 185 hastanın CMV reaktivasyonu sonuçları geriye dönük olarak incelendi. Nakille ilişkili standart parametrelerin yanı sıra HLA antijenleri arasındaki değişkenler de incelendi.

**Bulgular:** Major risk faktörü olarak adlandırılan alıcı (%100) ve vericilerdeki (%94,6) yüksek seropozitiflik oranlarına karsın bizim çalışmamızda reaktivasyon oranı %24,9 idi. Altta yatan hastalık, yaş, cinsiyet, hazırlama rejimi, hazırlama rejiminde anti timosit globulin (ATG) veya fludarabin kullanılmasının CMV reaktivasyonu üzerine etkisinin olmadığı görülmüştür. CMV reaktivasyonu graft versus host hastalığı (GVHH) olmayanlarla kıyaslandığında GVHH olan alıcılarda daha sık olarak bulunmuştur (p<0,0001). HLA-B14, HLA-DRB1*01, ve HLADRB1* 13 antijenleri olanlarda CMV reaktivasyonu daha sık (p<0,05) iken, HLA-A11, HLADRB1* 04 antijenleri olanlarda daha düşük bulunmuştur (p<0,05).

**Sonuç:** Tüm kök hücre alıcılarına uygulanabilecek CMV profilaksisi ve/veya tedavisi için oluşturulmuş evrensel bir risk skorlaması gerekmektedir. HLA antijenleri de dahil olmak üzere genetik risk faktörlerinin rolünün ortaya çıkarılması hücresel tedavi ve/veya aşılama yöntemleri gibi stratejilerin belirlenmesine olanak sağlayacaktır.

## INTRODUCTION

Despite the major advances in transplant techniques, reactivation of cytomegalovirus (CMV) remains a major cause of morbidity and mortality, particularly in allogeneic hematopoietic stem cell transplantation (AHSCT) recipients [1]. Defining the risk factors for reactivation will guide risk-adapted treatment strategies and avoid unconditional prophylactic pharmacologic agents that have risks and costs of their own. On the other hand, promising results with adoptive cellular therapy with CD8+ and CD4+ virus-specific T cells suggest that high-risk patients might benefit from such novel treatments [2,3,4]. Seropositivity of the recipient, reduced intensity conditioning, graft-versus-host disease (GVHD) above grade 2, and treatment with corticosteroids are among the factors previously reported to be associated with increased risk of CMV reactivation [5,6]. The prognostic factors and risk scoring systems defined so far do not apply to all AHSCT recipients. CMV reactivation occurs in up to 70% of seropositive recipients after AHSCT in the absence of antiviral prophylaxis [5,7,8,9]. Seropositivity for anti-CMV IgG antibody is very high in Turkey, varying between 96.4% and 97.8% [10,11]. Despite the very high rate of CMV seropositivity, the reactivation rate in AHSCT recipients is surprisingly much lower in Turkey compared to other countries around the world [7,9,12,13]. Genetic factors, whether human leukocyte antigen (HLA) or non-HLA factors, might be responsible for the discrepancies in reactivation rates in different parts of the world. However, the genetic determinants in the host and donor that might have a potential impact on reactivation remain obscure. Nevertheless, some HLA types have been previously reported to be associated with higher CMV reactivation rates in stem cell and solid organ transplantation recipients [12,14,15,16]. Single nucleotide polymorphisms of the genes of the immune system have also been previously shown to influence the incidence of GVHD and some infectious complications [17,18].

We investigated the role of various prognostic variables, including the HLA antigens of the donors and recipients, in posttransplantation CMV reactivation.

## MATERIALS AND METHODS

**Patients**

We evaluated the CMV infection status of 185 consecutive patients who received AHSCT for various hematological malignancies and severe aplastic anemia at the Stem Cell Transplantation Unit of Gazi University Hospital between September 2003 and December 2009. Patient characteristics are given in Table 1. HLA typing was performed or repeated for all the recipients and donors in the same laboratory. Serologic or molecular methods were used for assessment of HLA-A and HLA-B alleles, and molecular methods were used for HLA-DR alleles. Peripheral blood was the stem cell source in all transplants. Details of the conditioning regimens are shown in Table 1. Acute GVHD (aGVHD) was graded according to European Blood and Marrow Transplantation criteria [18]. Prophylaxis for GVHD consisted of cyclosporine and methotrexate in the myeloablative and cyclosporine and mycophenolate mofetil in the nonmyeloablative transplants. This study was approved by the local ethics committee of the Gazi University Medical Faculty.

**Definitions of CMV Reactivation and Disease**

CMV infection was defined as replication of CMV demonstrated in the body fluids or any tissue in the absence of clinical findings or organ involvement. CMV disease was defined as infection accompanied by clinical findings and/or the presence of organ involvement.

CMV DNA was routinely monitored once a week by quantitative PCR method (QIAGEN GmbH, Hilden, Germany). CMV reactivation was defined as 2 consecutive titers of CMV DNA above 500 copies/mL. CMV reactivations occurring in the first 100 days after AHSCT were designated as “early”, while “late” reactivations were reactivations that occurred after day +100.

As a risk scoring system, the CMV scoring index (CSI), defined recently, was also applied retrospectively to check whether this scoring system could be validated in our cohort [1]. According to this scoring system, the recipient’s CMV seropositivity conveyed a risk score of 4 points while donor CMV seropositivity was 1 point, presence of aGVHD was 2 points, and use of T cell-depleting agents was 1 point for antithymocyte globulin (ATG) and 2 points for alemtuzumab. The patients were stratified into 3 risk groups: low risk (0-2), intermediate risk (3-5), and high risk (6-7).

**Standards of Care for CMV Prophylaxis and Treatment**

All patients received leukocyte-filtered blood products irradiated by 3000 cGy of radiation. CMV reactivation was treated preemptively with ganciclovir, 5 mg/kg intravenously twice daily for 2 weeks, and then daily for 2 weeks until 2 consecutive negative PCR results were achieved.

**Statistics and Data Analysis**

Statistical analyses were performed using SPSS 15 for Windows. Patient characteristics, disease characteristics, and the association between the HLA type and CMV reactivation were compared using chi-square and Fisher’s exact tests for categorical variables. Other categorical risk factors were analyzed by chi-square and Fisher’s exact tests. Continuous and nonparametric variables were compared with the Mann–Whitney U test. Overall survival (OS) and progression-free survival (PFS) were estimated using the Kaplan–Maier method, with differences between patients groups with and without CMV reactivation compared by log-rank test. Logistic regression analysis was used to analyze the relevance of CMV reactivation and HLA type, steroid treatment, and aGVHD. Statistical significance was defined at p<0.05.

## RESULTS

**Patient Characteristics**

We studied 185 consecutive patients (119 males, 66 females) who received AHSCT for a variety of hematological malignancies and severe aplastic anemia. We retrospectively reviewed the medical records to assess the incidence of post-AHSCT CMV antigenemia and disease. Other variables that might influence the frequency of posttransplantation CMV reactivation, such as HLA antigens, conditioning regimens, presence of acute and chronic GVHD, presence of steroid treatment, or engraftment time, were also retrieved from the patients’ charts. Patients were followed for a median of 385 days after AHSCT. The characteristics of the patients included in the study are given in Table 1. 

One hundred and seventy-three patients received AHSCT from HLA-identical sibling donors and 12 patients received matched unrelated donor (MUD) stem cells. The CMV statuses of the patients and donors are shown in Table 2. All the recipients and 175 (94.6%) of the donors were CMV-seropositive. While all the Turkish sibling donors were seropositive, only 2 of the 12 MUD donors from overseas (Germany, USA) were CMV-seropositive.

Our data were reanalyzed according to a recently published risk scoring system to investigate whether this scoring system applies to our patient cohort as well [1]. One hundred and eight patients had intermediate risk (score of 3-5; 90.8%) while 17 patients had high risk (score of 6-7; 9.2%). There were no patients with a low risk score (score of 0-2). The incidence of CMV reactivation was as follows: 43 of 168 patients (25.6%) in the intermediate risk group and 3 of the 17 patients (17.6%) in the high risk group had at least 1 episode of reactivation (p>0.05).

**Incidence of CMV Reactivation and Disease**

A total of 46 subjects (24.9%) experienced CMV reactivation at a median of 56 days (range: 16-1038 days) after AHSCT. Twenty-nine of the 185 patients (15.7%) had early CMV reactivation while 17 patients (9.2%) had late CMV reactivation. Median time to early CMV reactivation was 48 days (range: 16-84 days). Median time to late CMV reactivation was 287 days (range: 124-1038 days). Eleven of the 46 patients (24.9%) developed recurrent reactivation episodes, with a total of 61 reactivation episodes.

CMV disease developed in 5 patients with reactivation (10.9% of the reactivated cases and 2.7% of the cohort), and 2 of these recipients succumbed to CMV disease (4.3% of the reactivated cases and 1.1% of the entire cohort). The data of 46 cases with CMV reactivation are given in Table 2.

**Engraftment and CMV Reactivation**

The engraftment days for neutrophils and platelets were similar in patients with or without CMV reactivation and recurrent CMV reactivation (p>0.05).

**GVHD and Steroid Therapy**

Ninety-one (49.2%) of 185 patients had GVHD after transplantation. Fifty-nine (31.9%) patients had acute while 32 (17.3%) had chronic GVHD. Thirty-two of the recipients with grade II-IV aGVHD and 6 with extensive chronic GVHD had active GVHD during CMV reactivation. Thirty-eight of the recipients, namely those suffering from active GVHD, were on concurrent steroid therapy. GVHD occurred at a median of 25 (range: 5-48) days after transplantation in cases with early reactivation and a median of 75 (range: 8-721) days after transplantation in cases with late CMV reactivation.

The groups with and without CMV reactivation were similar with respect to sex, diagnosis, underlying disease and its subtype and status, intensity and type of conditioning regimen, and the presence of T cell-depleting agents fludarabine and ATG in the conditioning regimen. CMV reactivation was similar in patients who were HLA-matched or mismatched by 1 or 2 antigens.

CMV reactivation rate was significantly higher in cases with GVHD compared to those without GVHD (p<0.0001). CMV reactivation was significantly more frequent in patients with aGVHD compared to patients with chronic GVHD (p<0.04). The frequency of CMV reactivation increased with ascending intensity of GVHD. CMV reactivation was significantly more frequent (p<0.0001) in cases with grade 3 aGVHD and diffuse chronic GVHD. The timing of CMV reactivation was a median of 27 (range: 5-48) days after GVHD in cases with early CMV reactivation. CMV reactivation was found to be significantly higher (p<0.0001) in patients with steroid-requiring GVHD.

In a subanalysis of patients with recurrent CMV episodes, 8 of the 41 patients (19.5%) with grade II-IV aGVHD or chronic extensive GVHD experienced repeating CMV episodes. The recurrent reactivation rate was 3 in 144 (2.0%) in patients without GVHD or with grade I aGVHD or chronic limited GVHD (p<0.001).

**Influence of Donor and Recipient HLA Type on CMV Reactivation**

The frequency of HLA antigens of the patients and donors was analyzed with respect to CMV reactivation. The frequency of each HLA antigen was analyzed and compared in patients with and without CMV reactivation (Table 3). CMV reactivation was significantly more frequent (p<0.05) in patients with HLA-B14, HLA-DRB1*01, and HLA-DRB1*13 antigens. On the other hand, CMV reactivation was significantly less frequent (p<0.05) in HSCT recipients with HLA-A11 and HLA-DRB1*04 antigens. 

**Figure 1:**
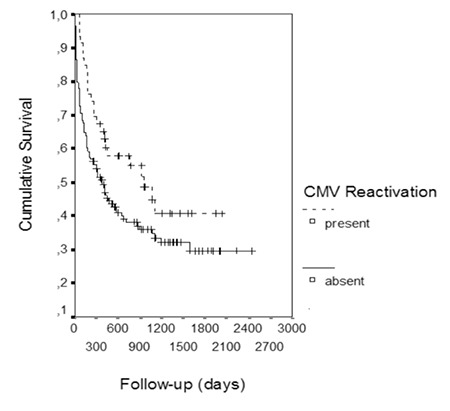
Overall survival in patients with and without cytomegalovirus reactivation.

Interestingly, survival was significantly longer in patients with CMV reactivation. The OS of the patients with CMV reactivation with a median follow-up of 516 days was 40.7% (median: 953 days), whereas the OS was 29.5% (median: 385 days) in patients without CMV reactivation with a median follow-up of 319 days (p<0.05; Figure 1). However, a subanalysis performed by excluding the very early deaths (20 patients who died within 30 days after transplantation) demonstrated similar survival in patients with and without CMV reactivation. The OS was 40.7% in patients with CMV reactivation with a median follow-up of 953 days, whereas OS was 34.5% in patients without CMV reactivation with a median follow-up of 546 days (p>0.05).

The PFS of patients with CMV reactivation was 52.9% (did not reach the median value), whereas the PFS was 46.2% (median: 529 days) in patients without reactivation (p>0.05).

In multivariate analysis including HLA-B14, HLA-DRB1*01, HLA-DRB1*13, aGVHD, and steroid treatment as variables, steroids were demonstrated to have an independent impact on CMV reactivation.

## DISCUSSION

CMV reactivation remains a cause of significant morbidity and mortality in AHSCT recipients. Risk factors should be identified to tailor the treatment. However, no scoring system that applies to all transplant recipients has been validated so far.

Various risk factors, including seropositivity of the donor and the recipient, type of conditioning, T cell depletion, donor source, and GVHD, have been claimed to be associated with CMV reactivation [19,20,21]. GVHD and the seropositivity of the donor and/or recipient are the common risk factors defined in most of these studies.

All of the recipients and sibling donors were CMV-seropositive in our series, while 17% of the unrelated donors were seropositive. The country of origin of the unrelated donors was the United States or Germany. Although CMV is endemic all over the world, its prevalence varies widely between 40% and 92.1% with respect to geographic and socioeconomic status of the pertinent country. The prevalence might vary even in different geographic parts of the same country [22,23,24,25,26]. The very high prevalence of seropositivity for CMV in donors and recipients in the present study is quite similar to previous reports on AHSCT recipients (97.2%) and the normal population (97.8%) in Turkey [12]. However, there is a major contradiction in our results with respect to previously established risk factors for CMV reactivation. Relatively low early and late reactivation rates were demonstrated in our patients despite the very high rate of seropositivity in the recipients/donors, which is also in accordance with the previous reports from Turkish transplant centers [13].

A risk scoring system, the CSI, was defined for CMV reactivation in AHSCT recipients in a recent multicenter report from Australia [1]. Their reactivation rates were 5.8%, 44.8%, and 67.7% in low, intermediate, and high risk groups, respectively. However, our data are far from verifying their results as the reactivation rate in our series was much lower. More than 90% of our patients had an intermediate CSI risk score with a 25.6% reactivation rate. More importantly, the reactivation rate was 17.6% in our patients with high CSI risk scores. Diverse reactivation rates in different countries using the same prognostic criteria need to be examined. Non-HLA genetic factors including single nucleotide polymorphisms, microsatellites of cytokines, and innate immunity have gained popularity over the last 15 years as factors that either contribute to or mitigate AHSCT complications [27]. We think that HLA or non-HLA genetic factors might be responsible for the discrepancies.

Thirty-three patients in our series received fludarabine in the conditioning regimen and this agent was not found to be a risk factor for reactivation, in contrast to some previous reports [1,28]. Ganciclovir causes delayed immune reconstitution in addition to its myelotoxicity, which suggests that it might have contributed to recurrent reactivation episodes in the 25% of our reactivated patients who received these agents in their initial reactivation episodes. However, the immunosuppressive effects of ganciclovir cannot solely explain the repeating episodes as some patients do not have repeating reactivations despite receiving the same treatment. We performed a subanalysis of patients with recurrent CMV reactivation. While 8 of the 41 patients (19.5%) with grade II-IV aGVHD experienced repeating CMV episodes, the recurrent reactivation rate was 3 in 144 (2%) in patients without aGVHD or with grade I aGVHD (p<0.01). Thus, GVHD seems to be the main predisposing risk factor for recurrent reactivation episodes.

GVHD was present in 49.2% of the patients presented in this study, approximately two-thirds of them having aGVHD. Eighty-three percent of the CMV reactivations were seen in patients with GVHD, while reactivation was more common in aGVHD compared to chronic GVHD. Steroid-requiring grade II-IV GVHD was found to be a significant factor contributing to CMV reactivation as 38 of the 41 patients with grade II-IV aGVHD had CMV reactivation at some point in the follow-up period of the study. Moreover, CMV reactivation was also correlated with the severity of GVHD. Almost all the relevant literature is in consensus with respect to the role of GVHD in CMV reactivation. There is also consensus on the cause of this association, namely the cellular and humoral immunodeficiencies related to GVHD itself and the drugs, particularly steroids, used in its treatment [29,30]. The role of antiviral CMV pharmacologic prophylaxis in patients with steroid-requiring GVHD could be the subject of prospective randomized trials. However, we should mention that the majority of the reactivations were successfully managed in our patient population.

Age, sex, conditioning regimen, and the degree of HLA matching did not have a significant impact on CMV reactivation. However, the relatively low number of HLA-mismatched transplants in the present cohort should be kept in mind. Neither fludarabine nor ATG in the conditioning regimen caused an increase in the reactivation rate, contrary to some previous reports [1,28]. We think that the decreased GVHD rate and immunosuppression leading to infectious complications caused by these agents might have balanced each other.

The mortality was 40% in patients with CMV disease in the presented series, which is unacceptably high, albeit much lower than in previously reported series [31]. Adoptive transfer of CMV-specific T cells for treatment, or donor and recipient vaccination strategies as a prophylactic modality in high-risk patients, might obviate the development of the disease and/or the mortality attributed to it [32,33]. This will only be possible by identifying patients at risk.

CMV reactivation was significantly more common in patients with the HLA antigens HLA-B14, HLA-DRB1*01, and HLA-DRB1*13, while it was significantly less common in patients with HLA-A11 and HLA-DRB1*04. The clinical relevance of these results is not clear. Some earlier reports have claimed that some HLA alleles, and HLA-DR15 in particular, might be associated with deficient immunity against CMV, although this was not verified later [16,34]. On the other hand, a higher incidence of HLA-DR15, HLA-A30, and HLA-B40 was found in a subgroup of AHSCT recipients with CMV reactivation in the absence of aGVHD [12].

Similar to our results, HLA-A11 was found to be associated with decreased frequency of CMV reactivation in liver transplant recipients [35]. The HLA-A11 antigen was also previously demonstrated to be associated with a robust cytotoxic T cell response against the Epstein–Barr virus, another latent viral infection [36,37,38].

Another intriguing result of the present study is that the OS of the patients with CMV reactivation was longer than that of those without reactivation. Findings of a survival advantage for patients with CMV reactivation have been published before [39,40]. The antigenic stimulus of CMV during viremia might have caused the clonal expansion of CMV-specific NK and T cells, which in turn might have led to a graft-versus-tumor (GvT) effect [41]. Indeed, the PFS of the patients with reactivation was longer at 52.9% (median not reached) than the PFS of the patients without reactivation of 46.2% (median: 529 days), although without statistical significance. Levels of CMV-specific CD8+ T cells have been demonstrated to be significantly higher in patients with reactivation. However, these cells were demonstrated to be less functional, with reduced cytokine production [42]. It should also be noted that the GvT effect is an integral part of GVHD, a transplant complication where CMV is significantly more frequent. However, subanalysis with the exclusion of the very early deaths in our cohorts demonstrated similar OS in the patients with and without CMV reactivation, suggesting that the patients who died very early did not live long enough to experience a CMV reactivation.

In conclusion, Turkish allogeneic transplant recipients in our study cohort had a relatively low rate of CMV reactivation despite the very high rate of CMV seropositivity in the donors and recipients. Our results failed to validate a previously defined scoring system, which suggests that ethnic, geographic, or genetic factors might contribute to CMV reactivation rate. CMV reactivation was significantly more common in patients with acute and chronic GVHD and the HLA antigens HLA-B14, HLA-DRB1*01, and HLA-DRB1*13, while it was significantly less common in patients with HLA-A11 and HLA-DRB1*04. The 40% mortality rate in the patients who developed CMV disease indicates the importance of risk-adapted prophylactic strategies. The role of genetic factors including HLA as possible risk factors warrants verification in large databases. 

**Conflict of Interest Statement**

The authors of this paper have no conflicts of interest, including specific financial interests, relationships, and/or affiliations relevant to the subject matter or materials included.

## Figures and Tables

**Table 1:**
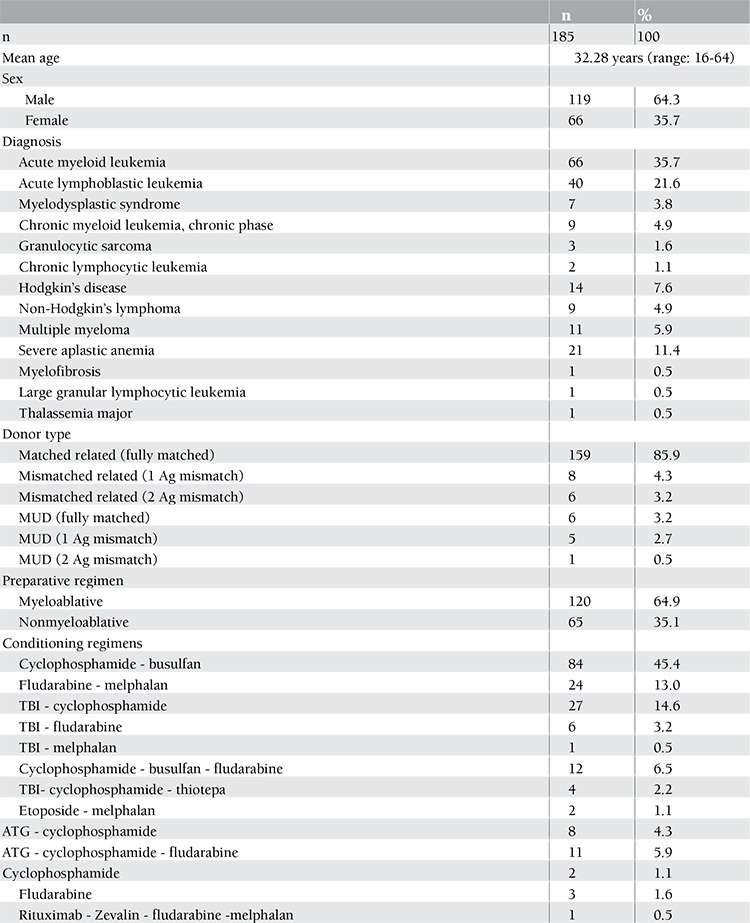
Patient characteristics.

**Table 2:**
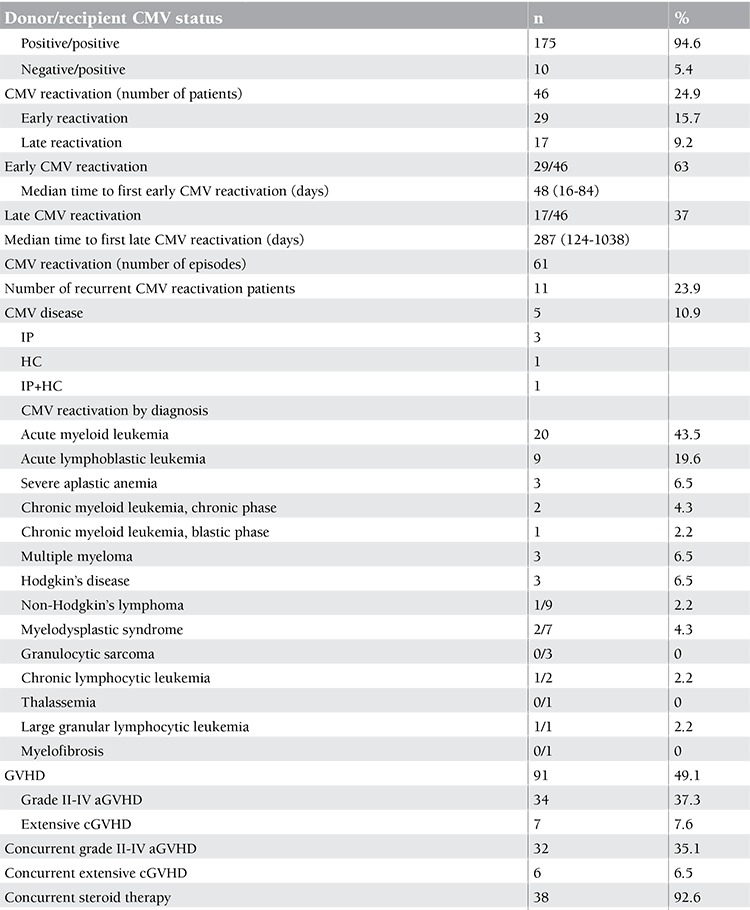
CMV reactivation in various risk groups.

**Table 3:**
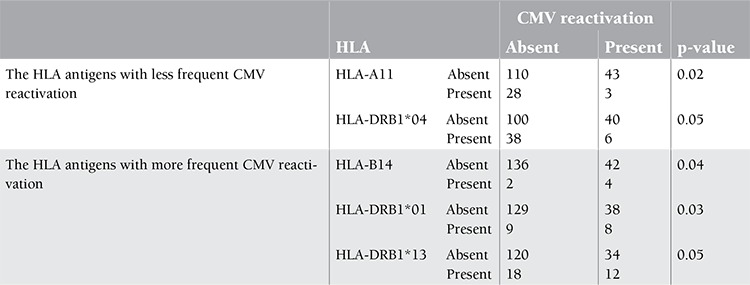
CMV reactivation and HLA-A status of patients and donors.
